# Revisiting the Fasciocutaneous Perforator Cross-Leg Flap

**Published:** 2016-04-28

**Authors:** Tom Reisler, David Buziashvili, Farrah C. Liu, Ramazi O. Datiashvili

**Affiliations:** ^a^Division of Plastic and Reconstructive Surgery, Department of Surgery, New Jersey Medical School, Rutgers University, Newark; ^b^New Jersey Medical School, Rutgers University, Newark

**Keywords:** cross-leg flap, cross-extremity flap, Hamilton, Ponten, fasciocutaneous flaps, free flap, lower extremity reconstruction

## DESCRIPTION

A 23-year-old man sustained an open pilon fracture of the ankle. Fractures were plated. A week later the medial wound dehisced with plate exposure. Good local tissue coverage was not available and free tissue transfer reconstruction was contraindicated because of lack of suitable recipient vessels, as demonstrated by computed tomographic angiography.

## QUESTIONS

1. **What are the reconstructive surgical options?**
2. **When was the first cross-leg flap described?**
3. **What are the principles behind a cross-leg flap?**
4. **What should the width to length ratio of cross-leg flap be?**

## DISCUSSION

Soft-tissue reconstruction of complex wounds in the distal third of the lower extremity always constitutes a challenge for surgeons. Surgical techniques vary according to the condition of the area to be reconstructed. Microsurgical free flaps are generally used but are not feasible when a suitable vessel is not available for microvascular anastomosis. It is therefore necessary to resort to other methods such as distally based islanded fasciocutaneous flaps from either the ipsilateral lower extremity or the contralateral leg (as a cross-leg flap).^1^^,^^2^ Cross-leg flap options include fasciocutaneous flaps,^3^^,^^4^ perforator fasciocutaneous flaps, perforator plus flaps,^5^ myocutaneous flaps,^6^ posterior tibial artery flap, and sural artery flap. The use of cross-extremity flap is particularly useful in situations, as in this case, where free tissue transfer cannot be employed and local methods cannot be used because of the extent of zone of injury as a result of a crushing injury.

The cross-leg flap dates back to 1854, when it was described by Hamilton to cure a chronic ulcer and after that it was successfully used for soft tissue coverage in the distal leg, especially during Second World War. After the introduction of microsurgery in 1970, pedicled cross-extremity flaps for lower limb wound coverage were replaced by free flaps, but in the aforementioned scenarios the cross-leg flap^4^ still has its role.

The cross-leg flap technique is a well-established method to cover soft tissue defects of lower extremity with exposed joints, tendons, bone, and metal hardware.^7^

Before Ponten, the cross-leg flaps were mere random pattern skin flaps without inclusion of deep fascia and with limited length-breadth ratio (1:1). To enhance the flap length, ‘delay’ was necessary, which increased the number of procedures and thereby hospitalization for several weeks. With the advent of fasciocutaneous flaps, described by Ponten in 1983, the cross-leg flaps have been raised safely and easily with width to length ratio of 1:3 to 1:3.5. This provided more room for movement between limbs avoiding cross-legging with minimal discomfort and inconvenience to the patient. Furthermore, in the late 1980s with an improved understanding of the vascularity of the soft tissue of the leg based on the perforators, fasciocutaneous flaps based on these perforators with a nonconventional dimension of more than 1:3 ratio were designed.^8^ As the positions of the perforators are almost always constant, they are reliable, and being perforator flaps, the pedicle can be narrowed, allowing more mobility and easy transfer. Several authors have advocated the routine use of external fixators in maintaining the position of cross-leg for ease of nursing care and postoperative wound management.

**Figure 1:**
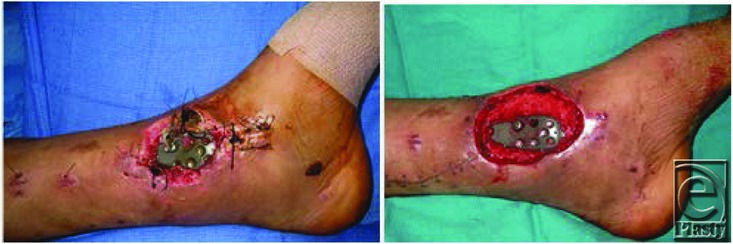
Pre- and postwound debridement.

**Figure 2:**
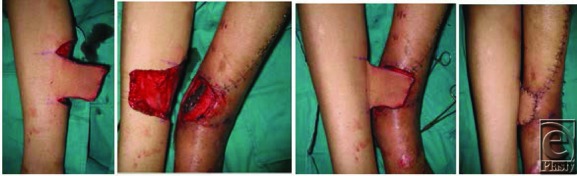
Raised medial fasciocutaneous perforator cross-leg flap, based on the posterior tibial artery perforators, and inset.

**Figure 3:**
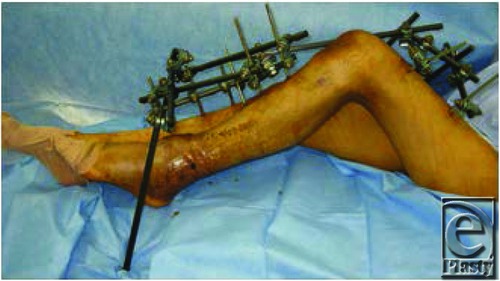
External fixator for maintaining the cross-leg flap in place.

**Figure 4:**
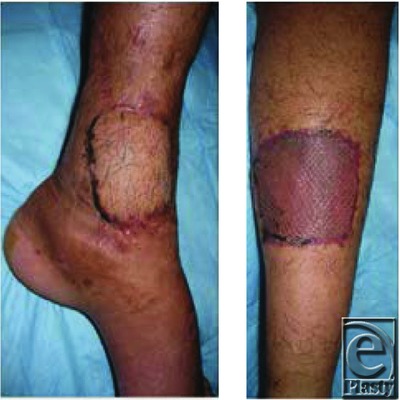
Three weeks' postoperative appearance recipient and donor site (covered with split skin graft), respectively.

The aforementioned patient underwent initial meticulous debridement and negative pressure wound therapy (Fig 1). Subsequently, the wound defect was covered with a medially based fasciocutaneous perforator cross-leg flap, based on the posterior tibial artery perforators (Fig 2). The perforators were identified preoperatively by a handheld Doppler, but no attempt was made to isolate or skeletonize the perforators when raising the flap. An external fixator was used to maintain the position of the cross-leg and for ease of nursing care and postoperative wound management (Fig 3). The pedicle was divided after 4 weeks and the donor site of the flap was split skin grafted (Fig 4). The patient resumed normal gait and activity without any stiffness of joints related with the flap or external fixator.
